# One new obligate commensal species of *Coenaletes* (Collembola, Coenaletidae) from the hermit crab *Coenobita compressus* (Crustacea, Coenobitidae) from Colombia

**DOI:** 10.3897/zookeys.1293.206618

**Published:** 2026-09-24

**Authors:** Angela Arango, Margarita Ojeda, Francisco Serna, José G. Palacios-Vargas

**Affiliations:** 1 Laboratorio de Ecología y Sistemática de Microartrópodos, Departamento de Ecología y Recursos Naturales, Facultad de Ciencias, Universidad Nacional Autónoma de México, 04510 Mexico City, Mexico Laboratorio de Ecología y Sistemática de Microartrópodos, Departamento de Ecología y Recursos Naturales, Facultad de Ciencias, Universidad Nacional Autónoma de México Mexico City Mexico; 2 Universidad Nacional de Colombia, Facultad de Ciencias Agrarias, Museo Entomológico UNAB, Grupo Sistemática de Insectos Agronomía (SIA), Bogota, Colombia Universidad Nacional de Colombia, Facultad de Ciencias Agrarias, Museo Entomológico UNAB, Grupo Sistemática de Insectos Agronomía (SIA) Bogota Colombia

**Keywords:** Key, new species, obligate commensal mites, ontogeny, seashore, springtails, taxonomy, teratology

## Abstract

A new species of *Coenaletes* associated with the hermit crab *Coenobita compressus* H. Milne-Edwards, 1836, living in shells of *Tegula atra* Lesson, 1830 (Gastropoda: Tegulidae) on the seashores of Chocó, Colombia, is described and illustrated. The ontogenetic development, including measurements and ventral setation from first instar to adult, is provided. A key for the identification of the three species known in the genus is included.

## Introduction

The study of microarthropods associated with hermit crabs has revealed different groups of mites and a few Collembola (Maldonado-Vargas and Palacios-Vargas 1999). Some mites parasitize several species of *Coenobita*, but the Coenaletidae (Collembola) are obligate commensals and exclusively associated with the terrestrial crab species. Only two species were known from this family of springtails: *Coenaletes vangoethemi* (Jacquemart, 1980) from New Guinea and *C. caribaeus* Bellinger, 1985 from Guadalupe Island, which was redescribed by Palacios-Vargas et al. (2000, 2026). We found a third species on the seashore of Chocó, Colombia, which is described here.

## Materials and methods

**Figures 1, 2.**
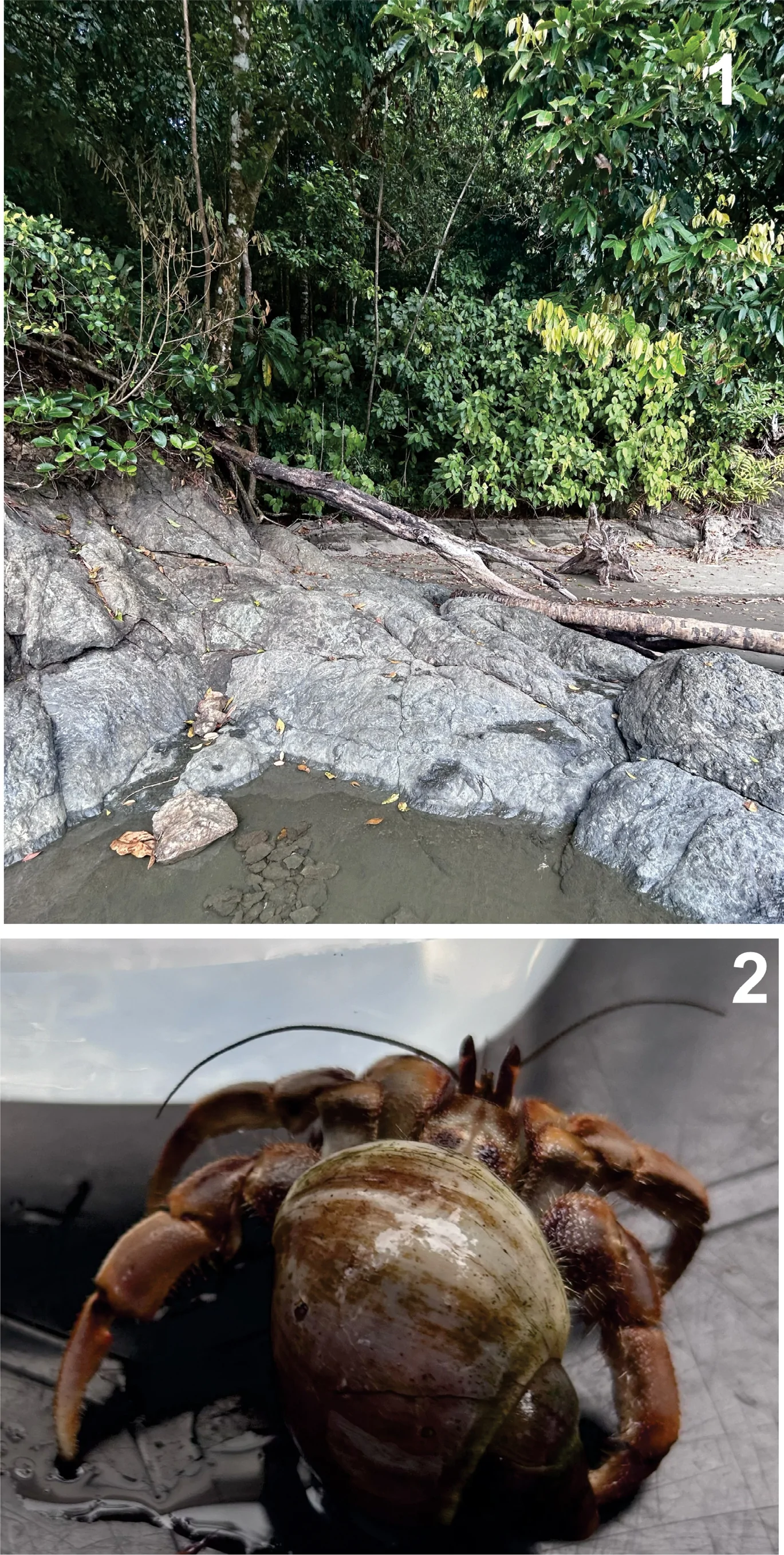
**1**. Type locality in Chocó, Colombia; **2**. Hermit crab *Coenobita compressus*.

An expedition was organized to the shores of Chocó, Colombia, to study the springtails living associated with hermit crabs (Fig. 1). Hermit crabs of the species *Coenobita compressus* H. Milne-Edwards, 1836 (Fig. 2) were placed in plastic containers with fresh water to induce the detachment of springtails and their exuviae, which then floated on the surface where they were collected. Specimens were fixed in 96% ethanol, then cleared with 10% KOH and lactic acid and mounted on slides using Hoyer’s solution. To harden the solution, the slides were dried on a slide warmer at 45–50 °C for one week. Finally, each slide was labeled with its data. Specimens were examined and photographed with a Carl Zeiss Primo Star phase-contrast microscope, and measurements were obtained. Several photographs were taken using a LEICA M205A stereomicroscope auto-mounting system and a HITACHI TM4000Plus II environmental scanning electron microscope. The specimens photographed using the auto-mounting system were transferred directly from the ethanol solution (in which they were preserved) into a 50:50 ethanol and glycerin solution inside in an open Petri dish. For SEM imaging, specimens were transferred from 70% ethanol to amyl acetate for five hours to maintain turgor and prevent body collapse within the vacuum chamber (Pérez-Benavides et al. 2023). Prior to SEM analysis, each specimen was air-dried and affixed with polyvinyl acetate adhesive to a cardboard micropoint mounted on one entomological pin.

Collection locality: COLOMBIA: Chocó, Nuquí, Coquí, Bocavieja, Safío Juan Salazar farm, 5°37'50.2"N, 77°19'47.6"W, 10 m asl, 2026.iii.31, J. G. Palacios, A. Arango, M. Ojeda & F. Serna, colls., *ex* hermit crabs *Coenobita compressus* (Decapoda: Coenobitidae) inhabiting shells of *Tegula atra* (Gastropoda: Tegulidae).

### Abbreviations:

**A** spine on Ant I;

**Abd** abdominal segment;

**Ant** antennal segment;

**B1–2** spines on Ant II;

**Bt** bothriotrichum, bothriotricha;

**C1–3** spines on Ant III;

**Cf** coxa furcalis;

**Ct** corpus tenaculi;

**Ext** external;

**Gp** genital plate;

**Interant** interantennal distance;

**Int** internal;

**L** length;

**M** macroseta;

**Mbt** microbothriotrichum;

**Me** mesoseta;

**ms** microsensillum, microsensilla;

**Post** postlabial setae;

**S** sensillum;

**Th** thoracic segment;

**Tita** tibiotarsus;

**W** width.

## Taxonomy

### Entomobryomorpha

**Coenaletidae Bellinger, 1985**

#### Coenaletes

Taxon classificationAnimaliaEntomobryomorphaCoenaletidae

Bellinger, 1985

59B692E2-07E4-526E-A561-D5E43728F0E2

##### Diagnosis (modified from Palacios-Vargas et al. 2026).

Mandible with molar plate but without apical teeth; maxilla small with six subequal lamellae. Pronotum reduced; Abd III and IV fused and elongated (dorsal and lateral, but not ventrally); other trunk segments distinct. Body tergites plurichaetotic, with ordinary setae conical and acuminate, varying in length. Differentiated macrosetae present dorsal and laterally on thorax and abdomen. With Bt, Mbt and ms on antenna and body. Bt on Abd II and III–IV, Mbt on trochanters and antennae. ms, one on each side laterally on Th II, and two pairs at end of Abd III–IV, close to the inner side of BtInt. Males with spines on Ant I–III, and on lateral tubercles of Th III and Abd III; dorsal apex of Tita excavate at base of unguis; pretarsal setae on papillae tubercles; unguis with enlarged lateral teeth, and capable of folding; furcula well developed, not reaching the ventral tube, and surrounding the big Ct, with dens smooth, bearing few setae; mucro falcate, fused to dens. Abd III and IV fused dorsally but not ventrally. Abd V and VI fused ventrally but not dorsally.

##### Type species.

*Coenaletes vangoethemi* (Jacquemart, 1980).

#### Coenaletes colombianus

Taxon classificationAnimaliaEntomobryomorphaCoenaletidae

Palacios-Vargas, Arango & Ojeda sp. nov.

C2C10B38-AC37-5B29-82BE-4D52133532B3

https://zoobank.org/F9FC889E-712A-4389-9782-21DBE8659A7A

Figs 3, 4, 5, 6, 7, 8, 9, 10, 11, 12, 13, 14, 15, 16, 17, 18, Tables 1, 2

##### Type material.

***Holotype***. • Male on slide: FC-UNAM:LESM-AC: 22877. ***Paratypes***: • two males, two females and 27 juveniles on slides. • One male, one female and 15 juveniles are kept at Facultad de Ciencias, UNAM. Slides numbered: FC-UNAM:LESM-AC: from 22878 to 22908. • One male, two females and 3 juveniles are kept at the Entomological collection of Museo entomológico UNAB, Facultad de Ciencias Agrarias, Universidad Nacional de Colombia, Bogotá, Colombia (UNAB 7222).

**Figures 3, 4.**
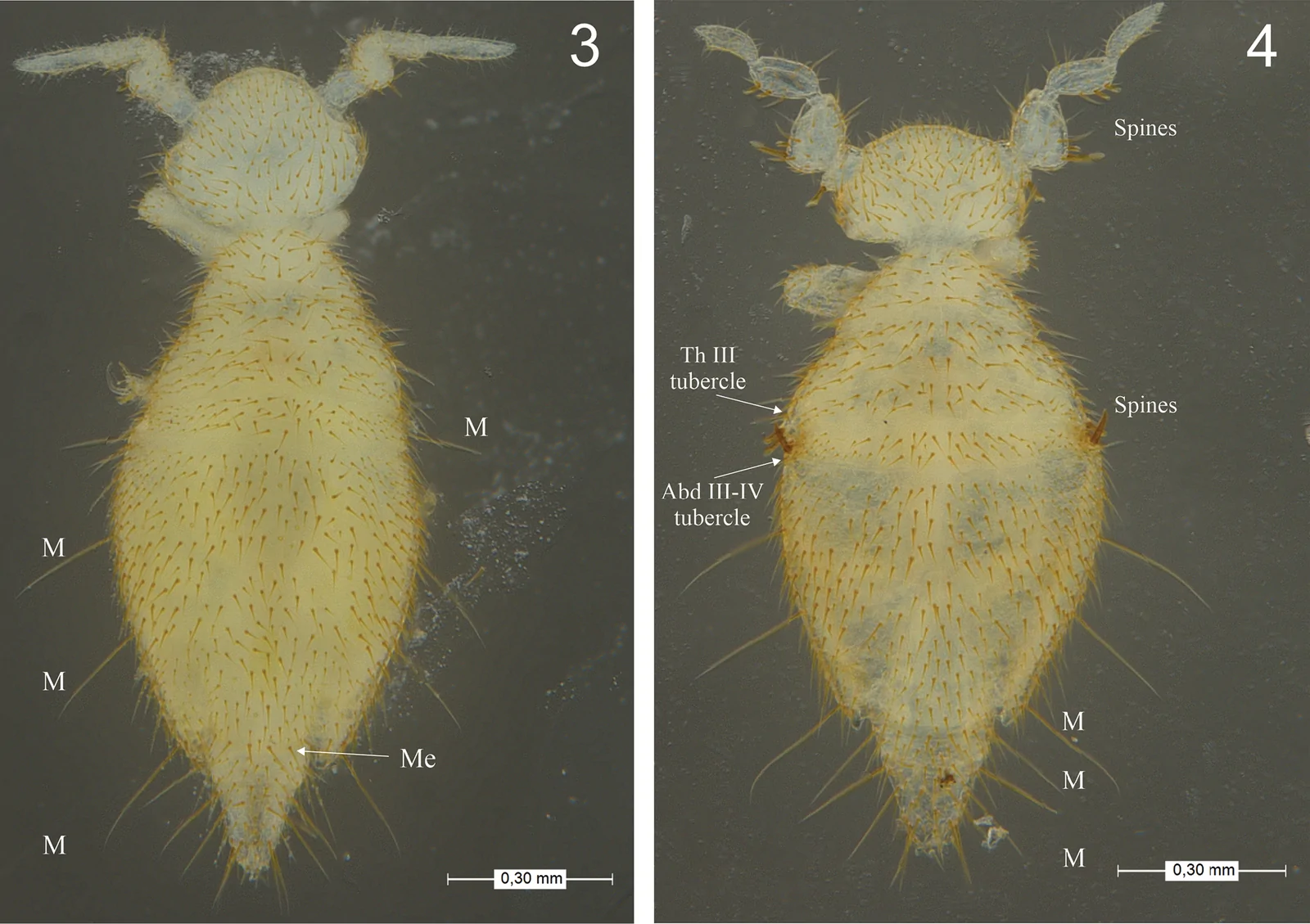
*Coenaletes colombianus* sp. nov. **3**. Habitus of female; **4**. Habitus of male, both dorsal view (images from stereoscopic microscope).

**Figure 5.**
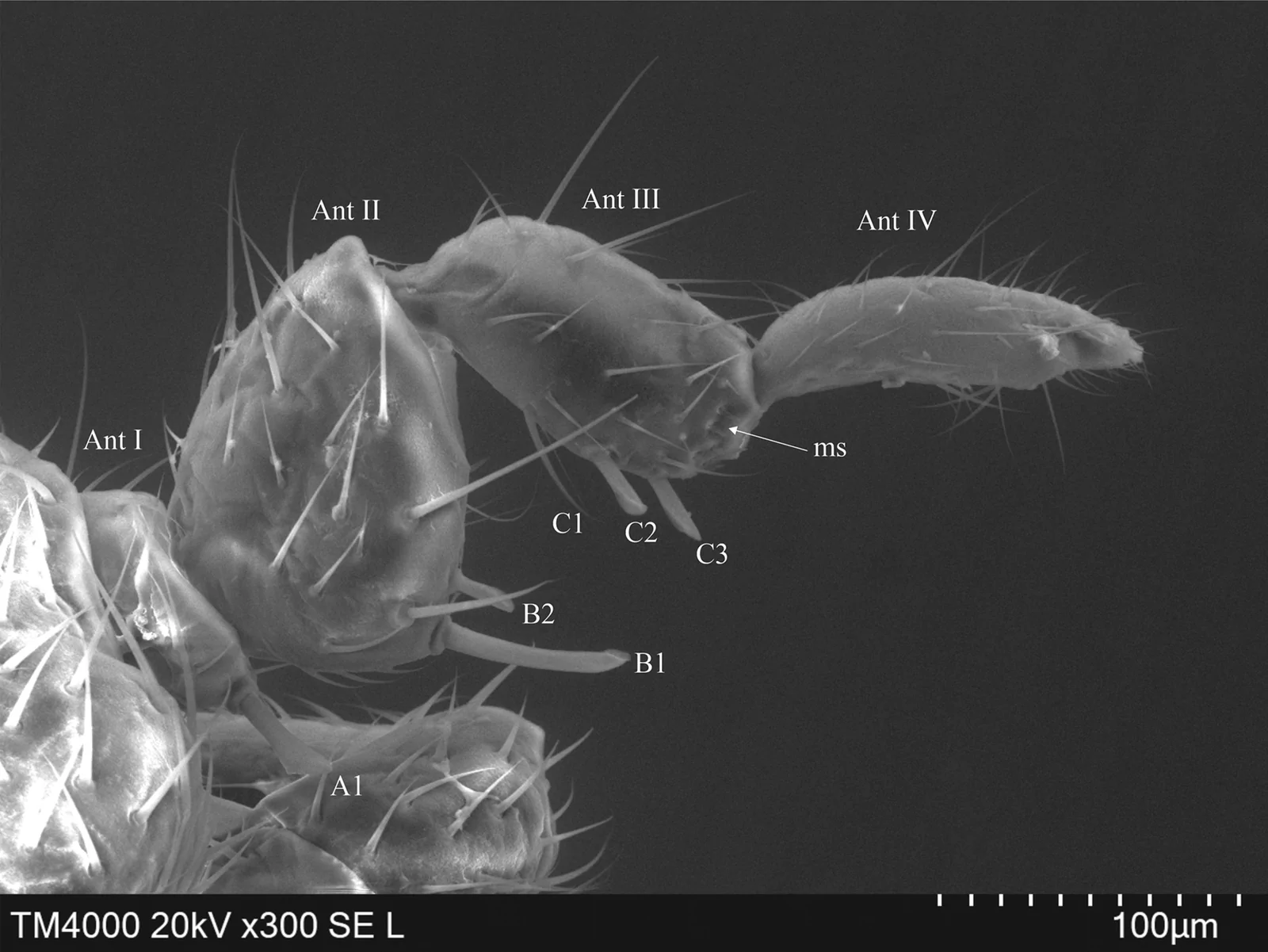
*Coenaletes colombianus* sp. nov. Ant I–IV of male, dorsal view under SEM showing A1, B1–2 and C1–3.

**Figure 6.**
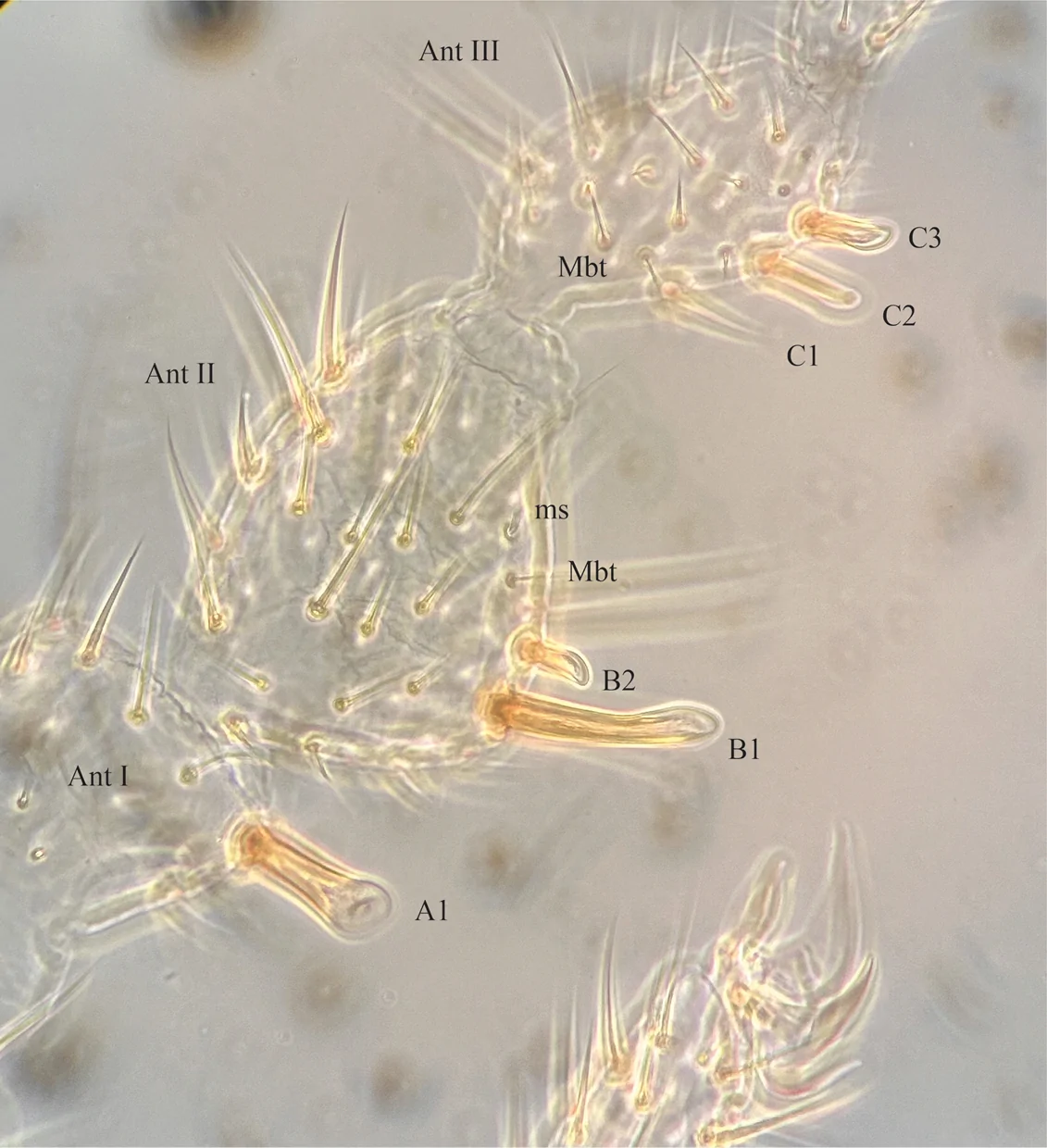
*Coenaletes colombianus* sp. nov. Ant I–III of male, view under phase contrast, showing Mbt, ms, and spines.

**Figure 7.**
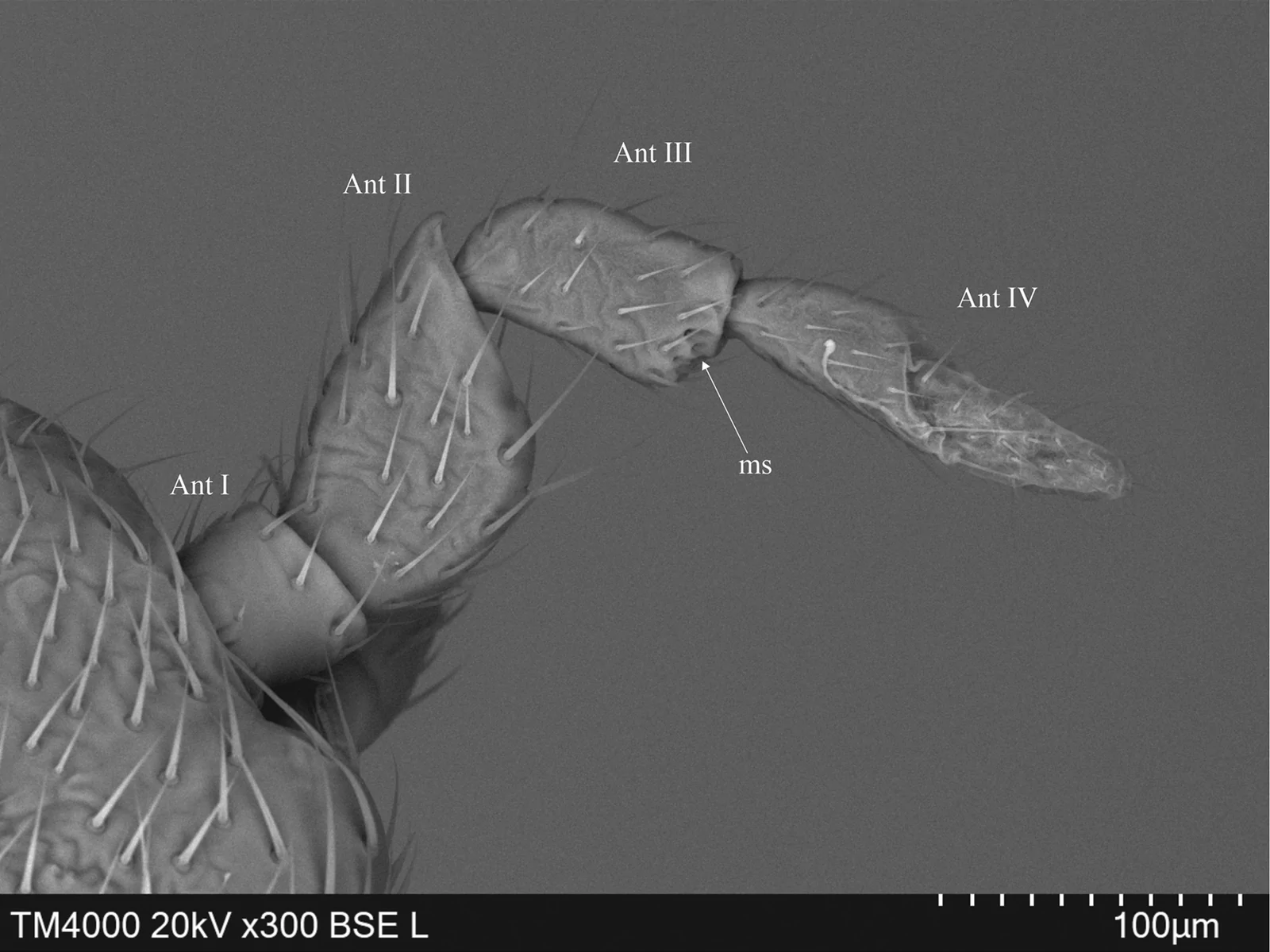
*Coenaletes colombianus* sp. nov. Ant I–IV dorsal view, under SEM.

**Figure 8.**
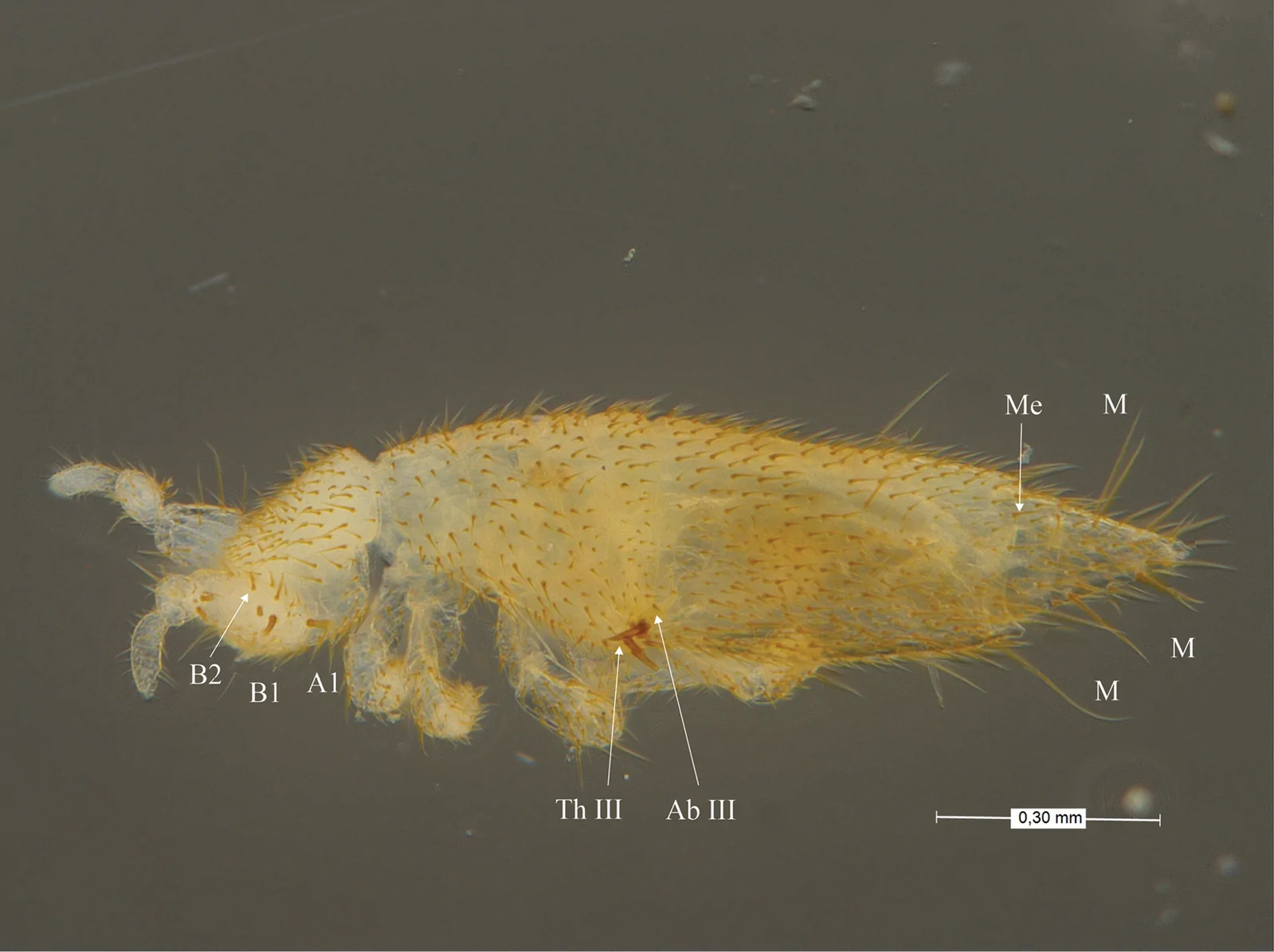
*Coenaletes colombianus* sp. nov. Lateral habitus of male (under stereoscopic microscope), lateral view showing spines, M and Me.

**Figure 9.**
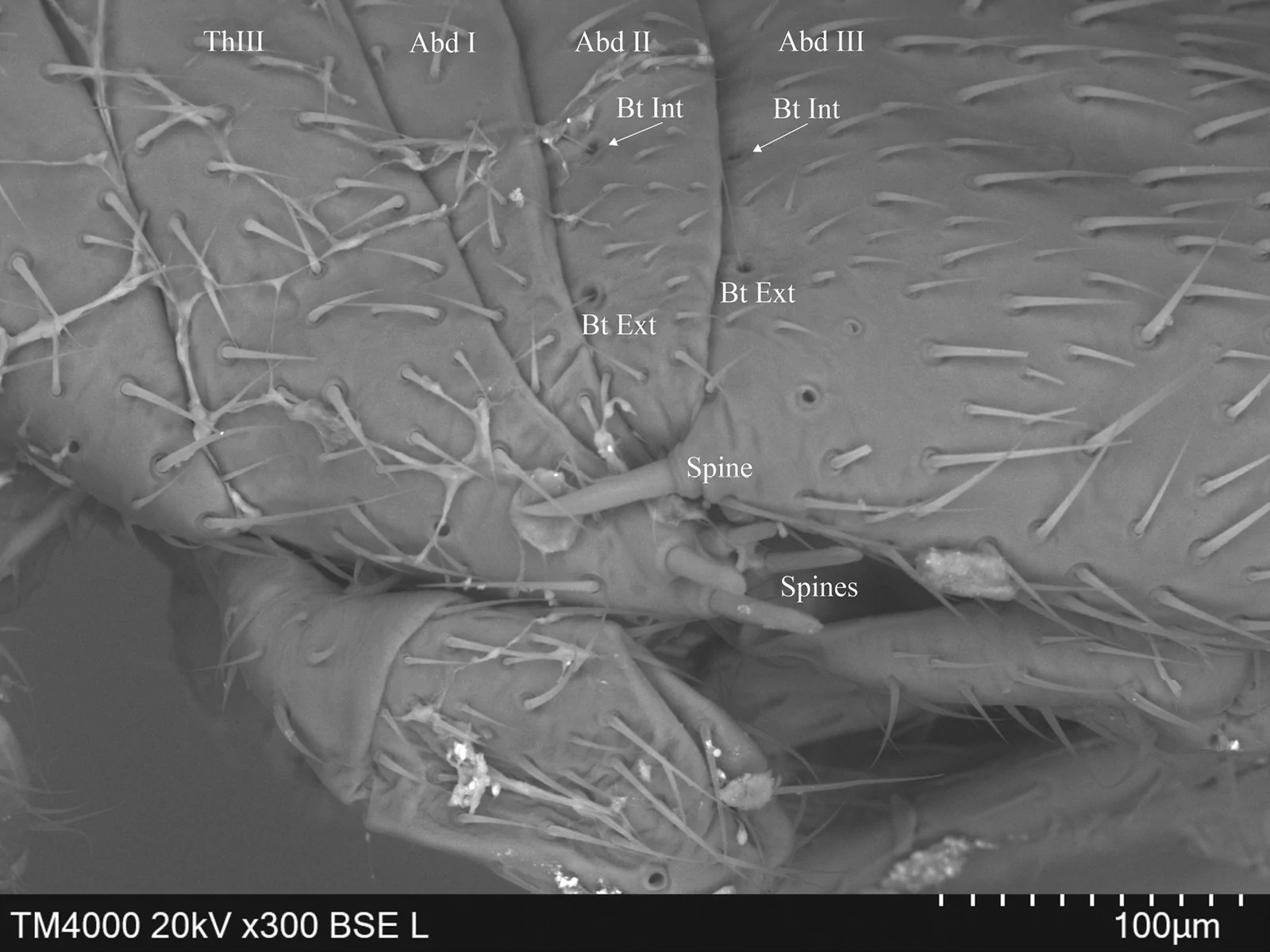
*Coenaletes colombianus* sp. nov. Lateral view under SEM of Th and Abd I–III, showing Th III tubercle with four spines and Abd. III–IV with a tubercle with one spine directed forward.

**Figure 10.**
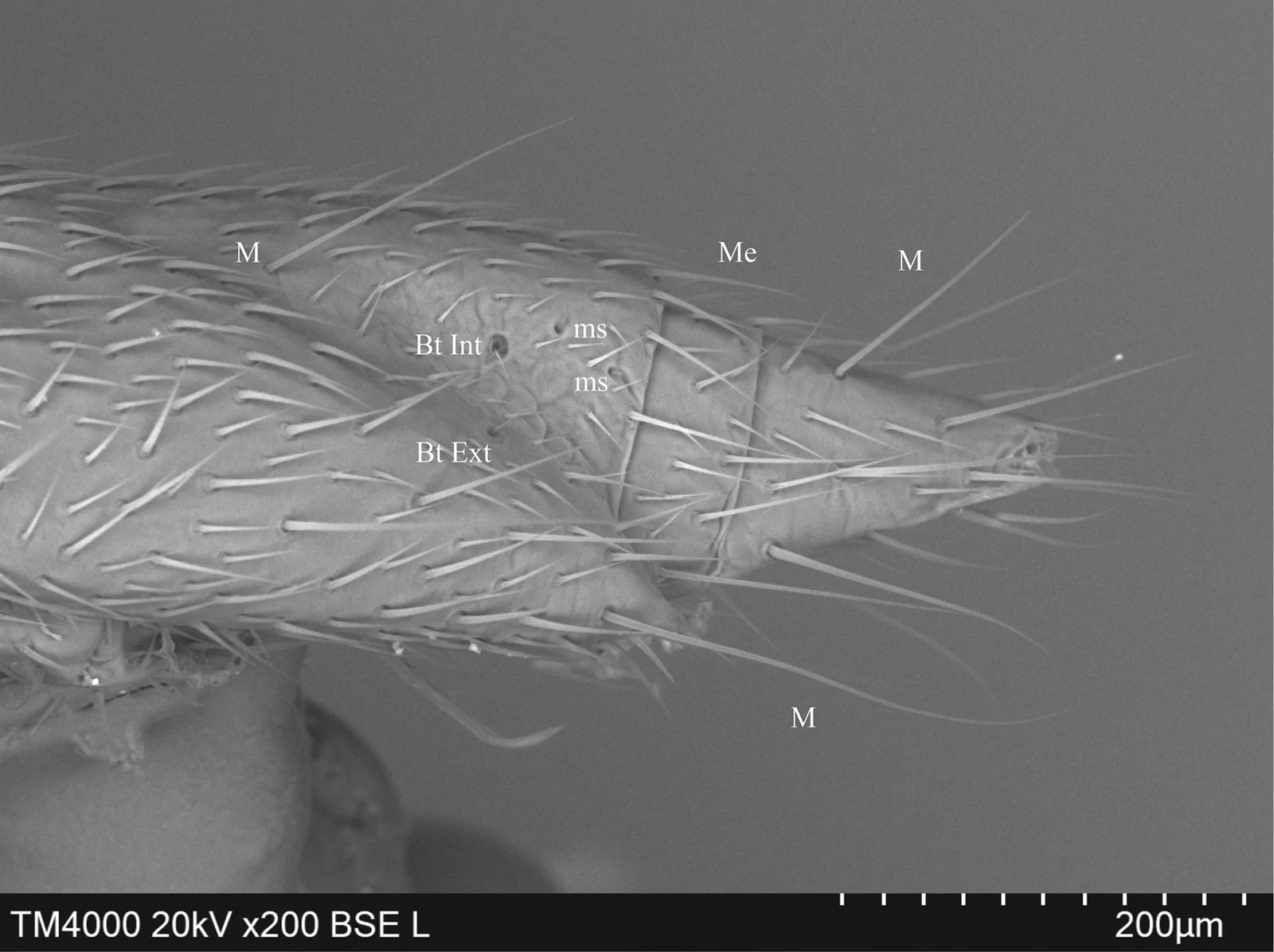
*Coenaletes colombianus* sp. nov. Lateral view under SEM of Abd III–VI, showing M, Bt, ms, Me.

**Figure 11.**
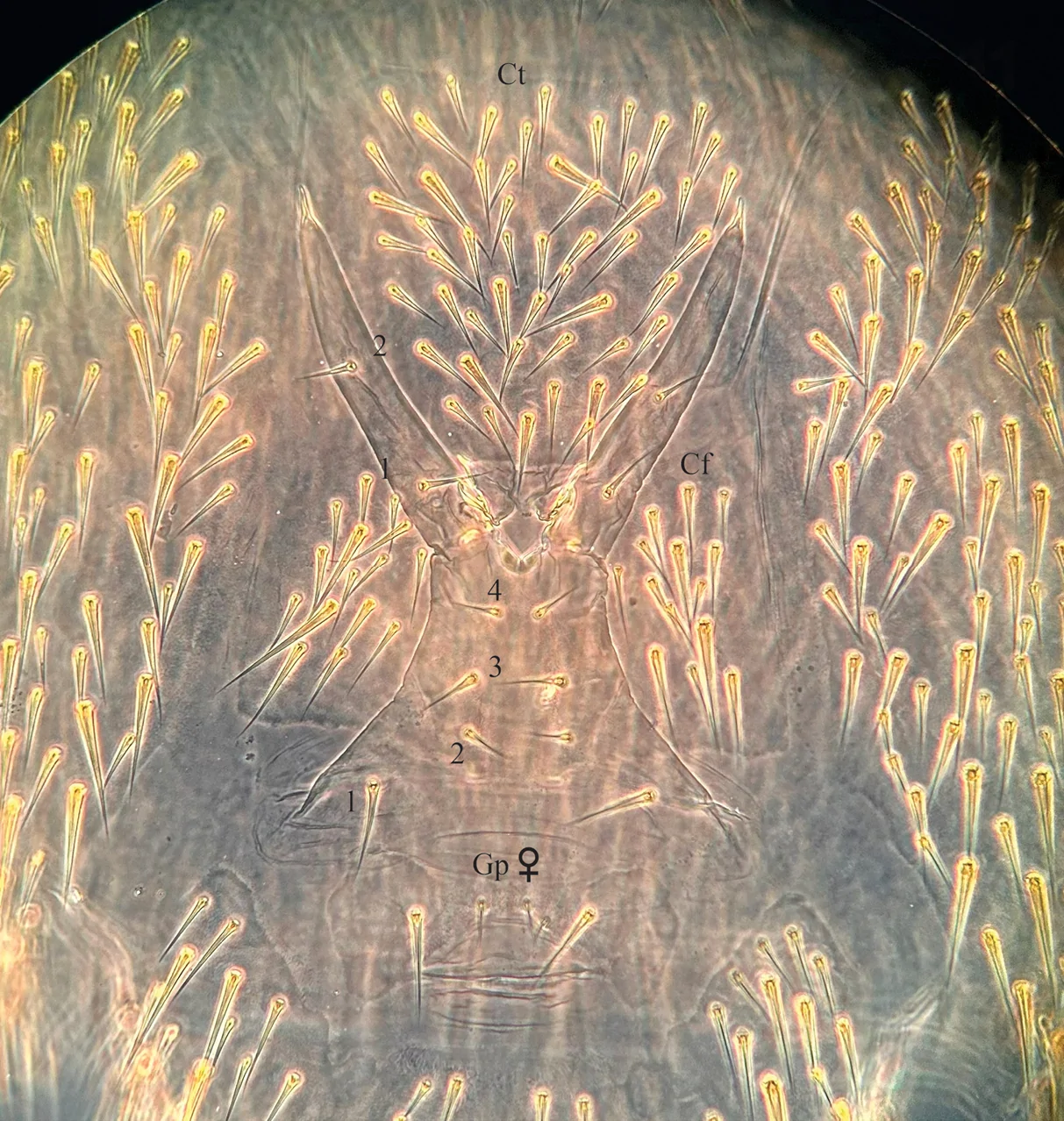
*Coenaletes colombianus* sp. nov. Ventral view under phase contrast, showing Ct furcula and female genital plate.

**Figure 12.**
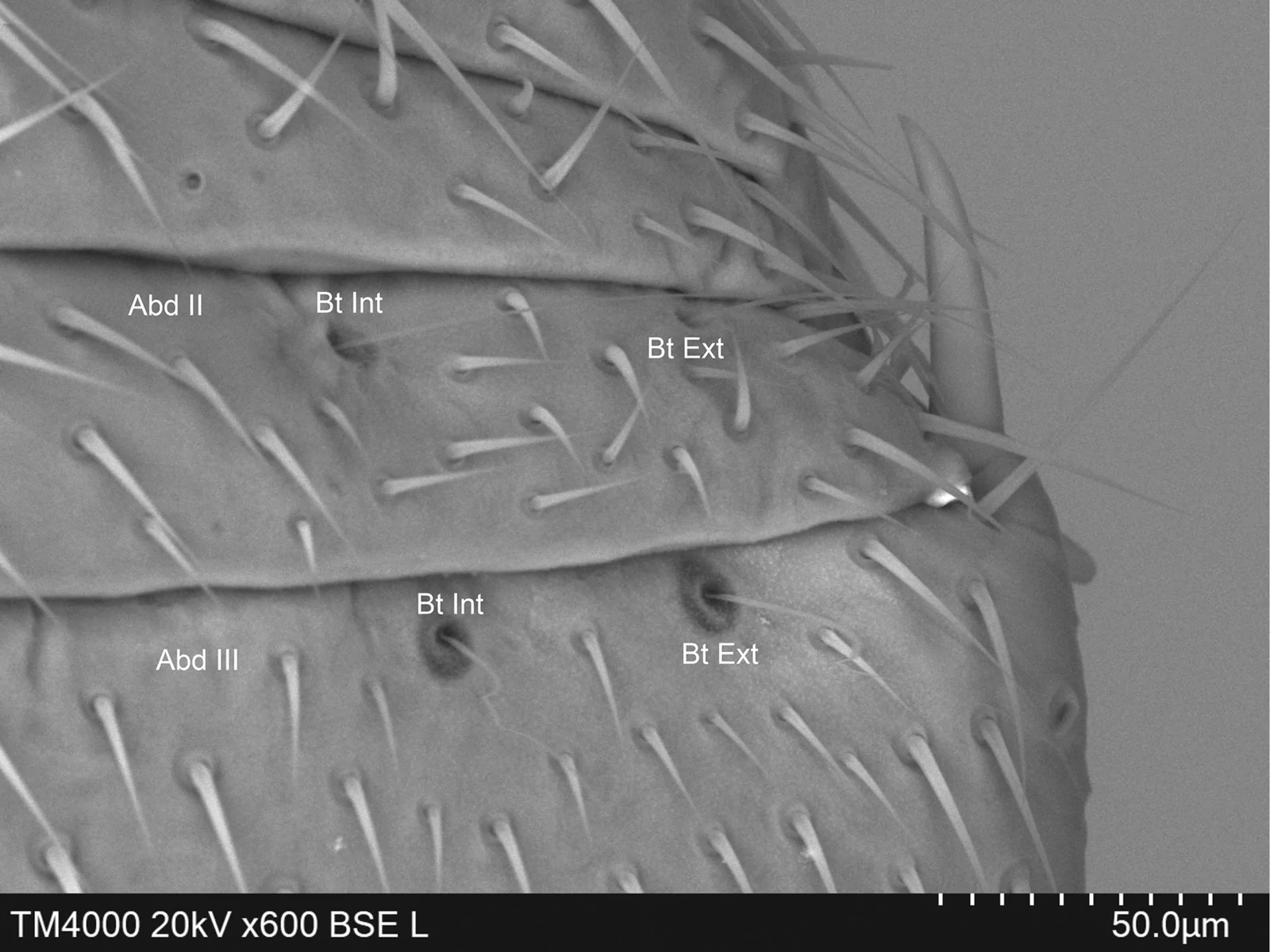
*Coenaletes colombianus* sp. nov., male under SEM, Abd I–III, dorsal view of right side, showing Bt In and Ext.

**Figure 13.**
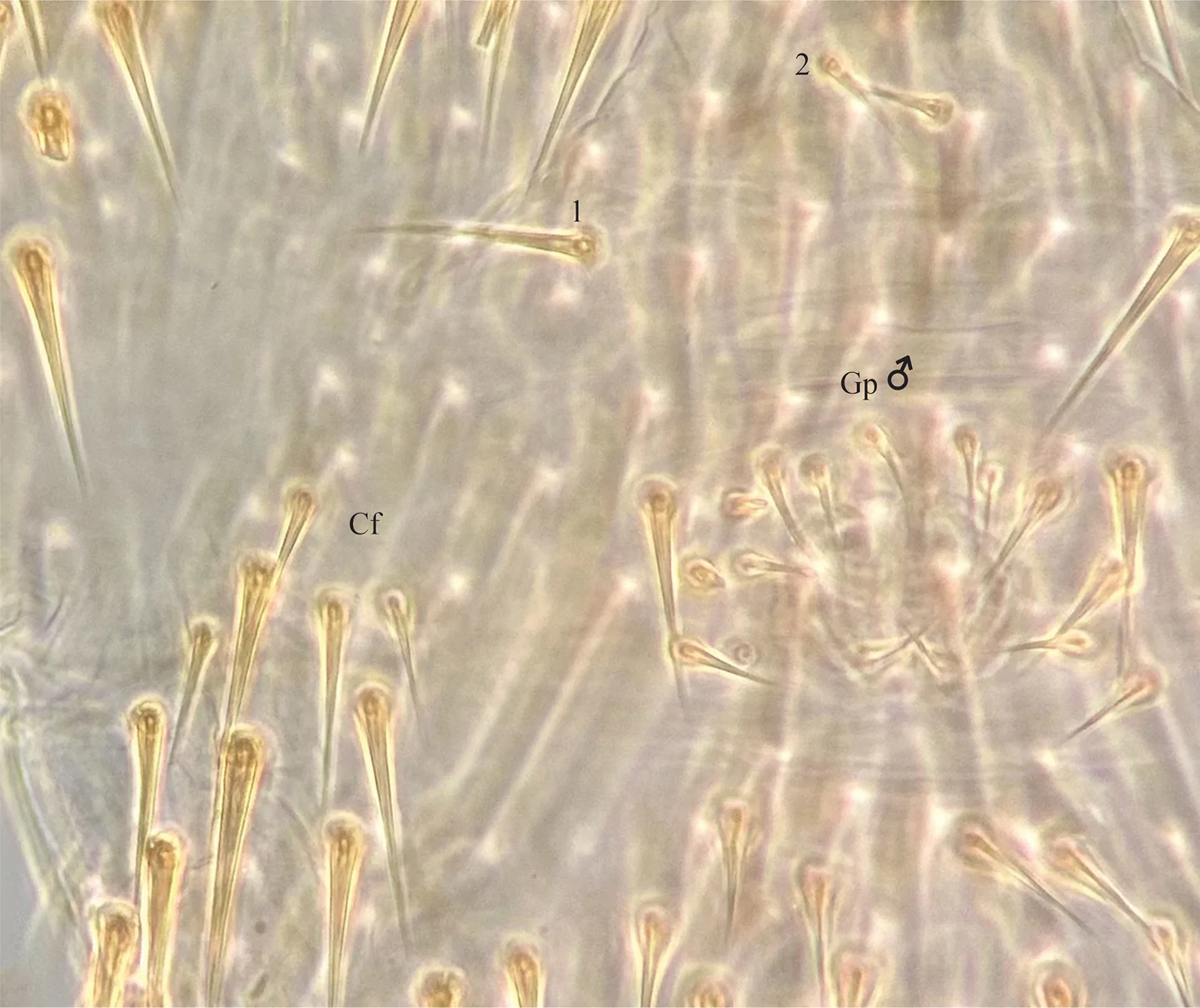
*Coenaletes colombianus* sp. nov. under phase contrast. Male genital plate, base of manubrium and Cf.

**Figure 14.**
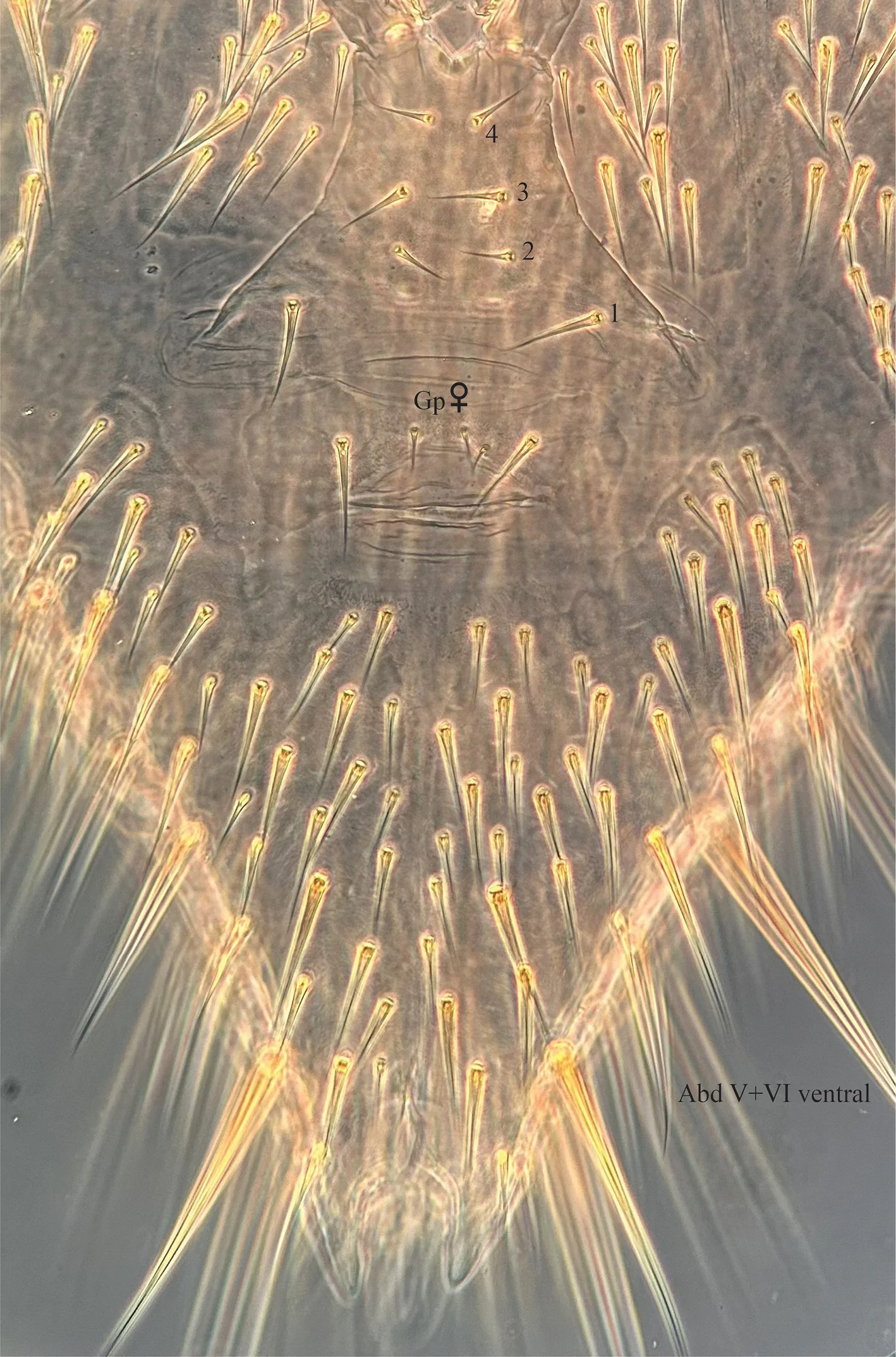
*Coenaletes colombianus* sp. nov. under phase contrast, ventral view Abd IV–VI, female genital plate, manubrium and the elongated Abd VI with lateral valve projections.

**Figure 15.**
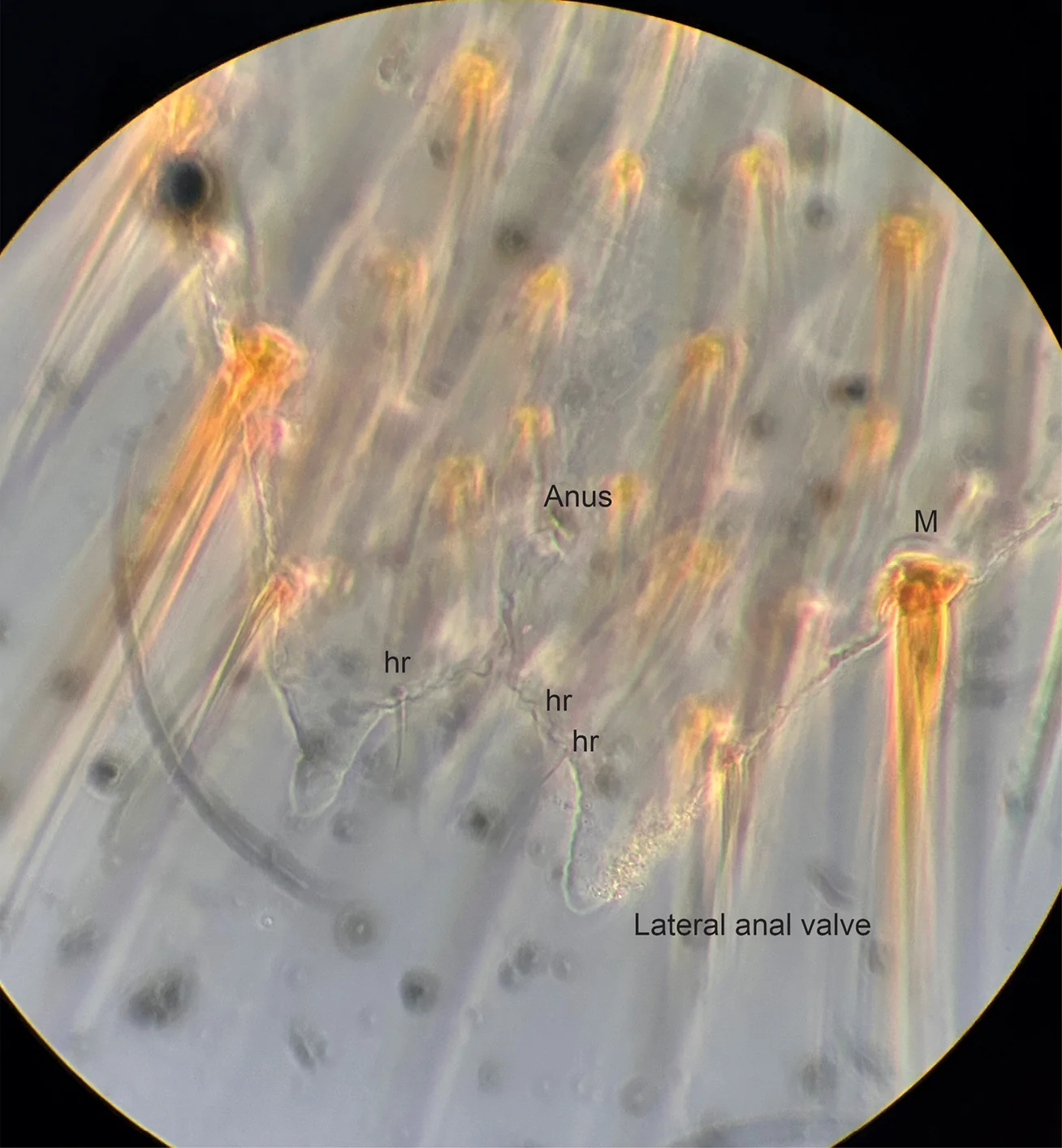
*Coenaletes colombianus* sp. nov. under phase contrast. Abd VI, ventral view showing setae hr on lateral anal valves.

**Figure 16.**
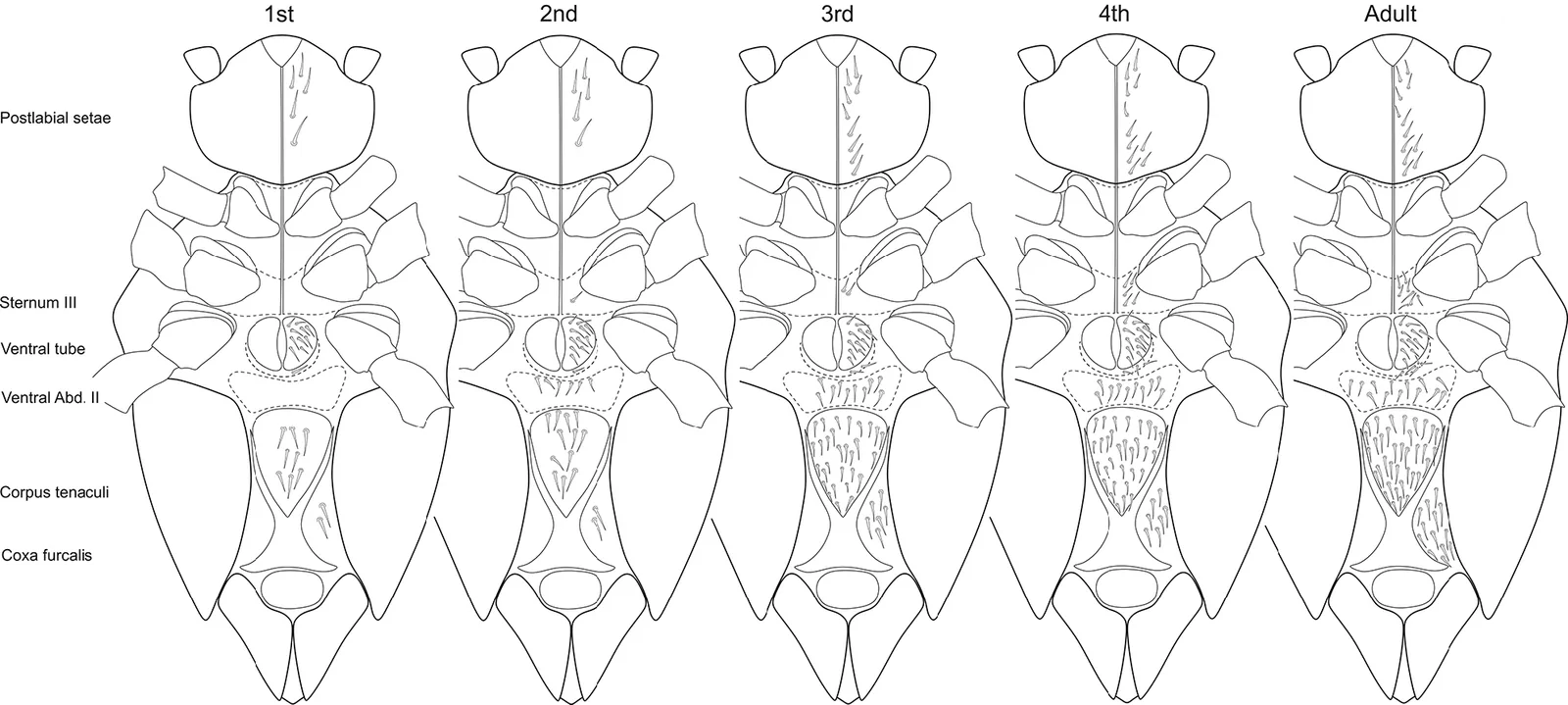
Ontogenetic series of *Coenaletes colombianus* sp. nov. showing the different postembryonic instars and adults.

**Figures 17, 18.**
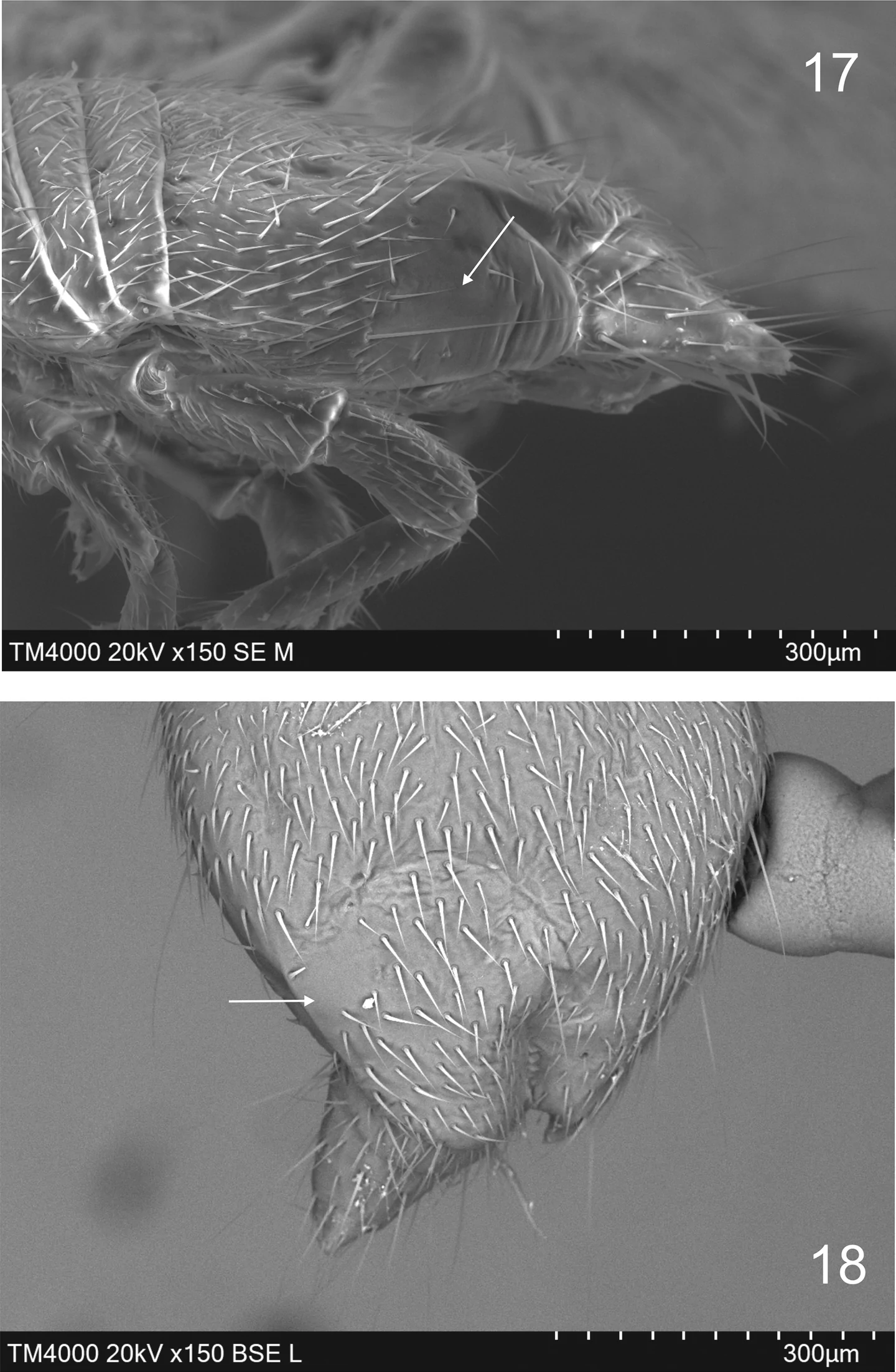
*Coenaletes colombianus* sp. nov. under SEM, with one teratological female. **17**. Lateral view of Abd I–VI; arrow indicates the area lacking normal chaetotaxy; **18**. Dorsal view of Abd III–IV to VI; arrow shows the distorted area.

**Table 1.** Measurements (in µm) of the ontogenetic development from first instar to adult males and females.

	**Instar I**	**Instar 2**	**Instar 3**	**Instar 4**	**male**	**female**
L	907	1.035	1.084	1.252	1.560	1.615
Head L × W	197 × 256	226 × 276	246 × 305	256 × 305	315 × 385	315 × 394
Interant	177	177	209	209	335	404
Ant I	42	47	50	55	79	72
Ant II L × W	72 × 57	92 × 62	107 × 72	110 × 72	160 × 111	138 × 84
Ant III	72	80	96	90	123	108
Ant IV	112	122	130	130	136	131
Tita I	132	150	162	162	193	190
Tita II	150	170	175	175	210	207
Tita III	180	192	210	220	268	287
Th II	89	148	108	108	197	168
Th III	79	79	99	89	118	128
Abd I	59	49	59	59	89	88
Abd II	49	59	69	69	99	78
Abd III–IV	246	305	354	355	493	492
Abd V	59	59	78	79	108	88
Abd VI	69	98	98	108	158	138
Mucrodens	110	127	142	123	178	153
BtAbd II	Int 190, Ext 270	Int 235, Ext 320	Int 265, Ext 362	271, 362	Int 385, Ext 525	Int 272, Ext 527
BtAbd III–IV Anterior	Int 180, Ext 270	Int 240, Ext 310	Int 282, Ext365	Int 283, Ext 381	Int 375, Ext 563	Int 390, Ext 512
BtAbd III–IV Posterior	Int 125, Ext 210	Int 175, Ext 220	Int 172, Ext 280	Int 175, Ext 343	Int 229, Ext 235	Int 239, Ext 379

**Table 2.** Comparison of ventral setation development of *C. colombianus* sp. nov. (variation in parentheses) from first instar to adult males and females.

	**Instar 1**	**Instar 2**	**Instar 3**	**Instar 4**	**Male**	**Female**
Post setae	5+5	5+5	9 (9–10)	11 (9–12)	11 (10–12)	12 (10–13)
Stern III	0+0	1+1	2 (1–3)	5 (3–5)	5 (5–6)	7 (5–9)
Ventral tube	10+1	11+1 (10–12+1)	11+2 (10–11+2–3)	11+ 3 (11–12+2–3)	11+5(11–12–5)	11+5 (10–11+2–3)
Abd II	0	6 (5–7)	10 (9–11)	10 (9–11)	17 (15–18)	14
Ct	8 (7–10)	11 (10–12)	27 (26–28)	30 (27–32)	41 (38–44)	47 (38–60)
Cf	2+2 (1–2)	2	4–9	6 (5–8)	10 (7–13)	13 (10–14)

##### Type locality.

Colombia – Chocó: Nuquí; ex. *Coenobita compressus*; Chocó, 5°37'50.2"N, 77°19'47.6"W, 10 m asl, 31 March 2026; J.G. Palacios-Vargas, M. Ojeda, A. Arango & F. Serna leg.

##### Diagnosis.

From 10+10 to 13+13 postlabial setae, retinaculum with 4+4 teeth and up to 60 setae on Ct, furcula only with posterior setae, 4 pairs on manubrium, mucrodens smooth, dens with 2 setae. Males with 1, 2, 3 spines on Ant I, II and III respectively.

##### Description.

Length of body 1.56 mm for males and 1.61 mm for females. Antennae of male 508 µm (*N* = 2), female 449 µm (*N* = 2) (Figs 3, 4). Head wider than long, male 315 × 385 µm ratio 1:1.2, female 296 × 375 µm ratio 1:1.06. Ant ratio (male) I–IV as 1: 2.0; 1.5; 1.7; female 1: 1.8; 1.6; 2.0. Ant II of male 160 × 111 µm much longer and thicker than that of female 138 × 84 µm (Table 1).

Ant IV with 2 subapical organs in separate minute pegs and six sensilla in apical half. Ant III with 8–9 sensilla of various sizes on outer apical face and 4 Mbt ventrally. Males with 3 different spines, C1 (38 µm), C2 (38 µm) and C3 (32 µm) with rounded and truncated apex. Ant II with 1 ms on outer apical face and 6 Mbt (Figs 5, 6) and spines of two different sizes B1, (73 µm) and B2 (25 µm), with truncate apex and minute serrations. Ant I with 9 setae in distal verticil and 5–6 more basal setae, 2 setulae and 1 thick spine A1 rounded apically (52 µm). Antenna of female identical to that of the male, except for the spines (Fig. 7) and relative length and width mainly on Ant II.

Interant distance in male 335 µm and in female 404 µm. Body depressed (Fig. 8). Mandible with a well-developed molar plate, lacking apical teeth. Maxilla with capitulum bearing six short, curved lamellae, finely fringed; maxillary outer lobe not discernible (as illustrated by Palacios-Vargas et al. 2026). Labrum with six small anterior labral setae and six longer prelabral setae. Labium with 4 setae on basomedial area, one longer and thicker; basolateral area with 5 setae; proximal area with 3 distal papillae, each bearing 1 apical and 1–2 subapical setae. Postlabial setae 12 (range 10–13) (Table 2).

Thorax with ms: 1+1 laterally on Th III, and 2+2 behind inner posterior Bt of Abd III–IV. Th III of males with 4 differentiated spines on a tubercle at each side, from smallest to biggest, A (30 µm); B (45 µm); C (45 µm); D (55 µm) (Fig. 9).

Abd I to III of males with 1 spine on each side, the last one upward, bigger, and sharp (Fig. 9). Length from I to III, 23 µm; 25 µm; 60 µm, respectively. M (in both sexes): 1+1 dorsolateral on Th III 100 µm; and Abd II 160 µm; 1+1 dorsally (140 µm) and 3+3 laterally on Th III–IV from 130 to 170 µm; 1+1 laterally on Abd V, 145 µm; 5+5 on Abd VI, 130–170 µm (Fig. 10). Anal valves cone-shaped and elongated with 2 hr setae each.

One Mbt on precoxa. Tita (I–III) with acuminate tenent hairs 3, 3, 4. Dorsal and posterior teeth of unguis forming lamellae comparable in size to unguis itself. Foot complex with unguiculus asymmetrical, identical to that described by Palacios-Vargas et al. (2026). Pretarsal setae on papillae. Sternal Th III with 5+5 to 9+9 setae. Ventral tube with 12+12 apical and 5+5 basal setae. Ct with about 60 setae and 4 teeth on each ramus. Sternum of Abd II with about 14–18 setae, Cf 7–14. Manubrium with 4 pairs of posterior setae, basal one longer than others, anterior surface with no setae (Fig. 11). Dens smooth with 2 posterior setae, distal one smaller. Mucro simple, fused to dens, about 1/9 of its length. Male genital opening with 10 (9–11) circumgenital setae, 2 pregenital setae and 2+2 (1+1) eugenital (Fig. 12). Female with 2 pregenital setae, 9–11 circumgenital and 1+1 eugenital (Figs 13, 14).

Anus opening at the end of Abd VI (Fig. 15), showing the lateral anal valves and the dorsal elongated ones. Specimens show a small concavity in the middle of Abd III–IV (Fig. 10) and a posterior lateral projection of the fused segments. Posterior part of these fused segments with 6 equidistant mesosetae, 100 µm long.

##### Ontogenetic development.

Because of the hypertrichosis, the dorsal setal pattern is not possible to check during development. Ventral changes are clearer; the gradual increase in the number of setae on the ventral side of the body during ontogeny shows that the most important changes are in Ct, Th sternum III, ventral Abd II, Cf and postlabial setae (Fig. 16). The ventral tube has few changes, mainly in the posterior setae. Table 1 gives all measurements in µm from the first instar to adults. The number of ventral setae and variation (in parentheses) are given in Table 2.

##### Etymology.

The specific epithet colombianus is a geographical adjective in the masculine nominative singular, referring to Colombia, the country where the type material was collected.

##### Distribution.

*Coenaletes colombianus* sp. nov. is only known on shores of Chocó, Colombia.

### Key to the species of *Coenaletes*

**Table:** 

1	Males with antennal spines (from Ant I to III) 1, 2, 3	**2**
–	Males with antennal spines (from Ant I to III) 2, 2, 3; lateral spines on the trunk (Th III and Abd I to III) 4, 1, 1, 1; dens smooth with 3 posterior setae in basal half	***C. caribaeus* Bellinger, 1985** Caribbean Ocean (Guadalupe Island, Dominican Republic and Mexican Caribbean)
2	Dens smooth with 2 posterior setae along the appendix; lateral spines on the trunk (Th III and Abd I to III) 3, 1, 2, 1	***C. vangoethemi* (Jacquemart, 1980)** (New Guinea)
–	Dens smooth with 2 posterior setae along the appendix; lateral spines on the trunk (Th III and Abd I to III) 4, 1, 1, 1	***C. colombianus* sp. nov. (**Colombia, Chocó, Pacific Coast)

## Discussion

The genus *Coenaletes* has many stable generic characters, and the species differ little. The main difference between *C. colombianus* sp. nov. and *C. caribaeus* Bellinger, 1985 is that the new species has only two setae on the dens, similar to *C. vangoethemi*. The number of antennal spines (from Ant I to III) in *C. caribaeus* is 2, 2, 2, whereas in *C. vangoethemi* it is 1, 2, 3, and in *C. colombianus* sp. nov. it is also 1, 2, 3. The lateral spines on the trunk (Th III and Abd I to III) in *C. colombianus* sp. nov. are 4, 1, 1, 1, as in *C. caribaeus*, whereas in *C. vangoethemi* they are 3, 1, 2, 1. In terms of total body length, *C. vangoethemi* is the smallest, but there are no significant differences between *C. caribaeus* and the new species. The head of both males and females is much wider than long in the new species compared to the other two, which also results in a greater interantennal distance in the new species. The mucrodens is also longer in males of the new species (178 vs. 121 µm) than in *C. caribaeus*.

There are important ventral characters that were omitted in previous descriptions of the two species known from this commensal springtail, such as (1) postlabial chaetotaxy, (2) ventral setation of Th III, (3) ventral setation of Abd II, and (4) coxa furcalis. They vary during ontogenetic development, as can be seen in *C. colombianus* sp. nov. (Table 2 and Fig. 16). *Coenaletes vangoethemi* (based on the original description drawings) has 16 setae on the ventral Abd II, 31 setae on the corpus tenaculi, and 17 setae on the coxa furcalis. One character not previously analyzed is the width-to-length ratio of Ant II. In *C. colombianus* sp. nov., it is 1.44, whereas in *C. caribaeus* it is 1.26. Thus, Ant II is thicker in the new species. The other antennal segments do not show strong differences. There are no important differences in the size of the thoracic spines between the two species.

### Variation

There are many asymmetries in body setation, as seen during ontogenetic development ventrally (see Table 2), but dorsally some specimens lack M on Abd III–IV. One teratological specimen was found, which lacked setae laterally on Abd I–III; on the left side of Abd III–IV most ordinary setae are lacking: all M, both Bt, ms, and most Me (Fig. 17). The abdomen is deformed on Abd III–IV (Fig. 18).

## Supplementary Material

XML Treatment for Coenaletes

XML Treatment for Coenaletes colombianus

## Additional information

### Artificial Intelligence (AI) use

The authors accept full responsibility for the content of the manuscript, including the disclosure of any use of AI.

No AI tools were used in the preparation of this manuscript.

### Author ORCIDs

A. Arango https://orcid.org/0000-0003-2873-8309

M. Ojeda https://orcid.org/0000-0001-6430-3455

F. Serna https://orcid.org/0000-0002-6143-9821

J.G. Palacios-Vargas https://orcid.org/0000-0001-9097-6813

### Data availability

All of the data that support the findings of this study are available in the main text.
